# The crosstalk between the Notch, Wnt, and SHH signaling pathways in regulating the proliferation and regeneration of sensory progenitor cells in the mouse cochlea

**DOI:** 10.1007/s00441-021-03493-w

**Published:** 2021-07-05

**Authors:** Jingfang Wu, Wen Li, Luo Guo, Liping Zhao, Shan Sun, Huawei Li

**Affiliations:** 1 NHC Key Laboratory of Hearing Medicine, ENT Institute and Department of Otorhinolaryngology, Eye & ENT Hospital, Fudan University, Shanghai, 200031 China; 2 State Key Laboratory of Medical Neurobiology and MOE Frontiers Center for Brain Science, School of Basic Medical Sciences, Fudan University, Shanghai, China; 3 The Institutes of Brain Science and the Collaborative Innovation Center for Brain Science, Fudan University, Shanghai, China

**Keywords:** Ototoxicity, Cell regeneration, Sensory progenitor cells, Signal pathway

## Abstract

Sensory hair cells (HCs) are highly susceptible to damage by noise, ototoxic drugs, and aging. Although HCs cannot be spontaneously regenerated in adult mammals, previous studies have shown that signaling pathways are involved in HC regeneration in the damaged mouse cochlea. Here, we used a Notch antagonist (DAPT), a Wnt agonist (QS11), and recombinant Sonic hedgehog (SHH) protein to investigate their concerted actions underlying HC regeneration in the mouse cochlea after neomycin-induced damage both in vivo and in vitro. With DAPT, the numbers of HCs increased, and supporting cell (SC) proliferation was seen in both the intact and damaged cochlear sensory epithelia, while these numbers were unchanged in the presence of QS11. When simultaneously treated with DAPT and QS11, the number of HCs increased dramatically, and much greater SC proliferation was seen in the cochlear epithelium. In transgenic mice with both *Notch1* conditional knockout and *β-catenin* over-expression, cochlear SC proliferation and HC regeneration were more obvious than in either *Notch1* knockout or *β-catenin* over-expressing mice separately. When cochleae were treated with DAPT, QS11, and SHH together, SC proliferation was even greater, and this proliferation was seen in both the HC region and the greater epithelial ridge. High-throughput RNA sequencing was used to identify the differentially expressed genes between all groups, and the results showed that the SHH and Wnt signaling pathways are involved in SC proliferation. Our study suggests that co-regulation of the Notch, Wnt, and SHH signaling pathways promotes extensive cell proliferation and regeneration in the mouse cochlea.

**Supplementary Information:**

The online version contains supplementary material available at 10.1007/s00441-021-03493-w.

## Introduction

The mammalian cochlea has very limited spontaneous hair cell (HC) regeneration ability, and such regeneration is only seen at embryonic or very early neonatal ages and the quantity and quality of this limited spontaneous HC regeneration cannot restore cochlear function (Jung et al. 2013). When the inner ear sensory HCs are damaged, there is no spontaneous HC regeneration in the adult cochlea, and this leads to permanent hearing loss (Herranen et al. 2020; Nakano et al. 2020). Thus, stimulating sensory precursor cell proliferation and transdifferentiation into new HCs might be an effective method for rescuing hearing dysfunction (Yamoah et al. 2020).

Signaling pathways play fundamental roles in biological activities, and many signaling pathways have crosstalk with each other, which is also the case in the period of inner ear development and during HC regeneration (Jayasena et al. 2008; Petrovic et al. 2015; Roccio and Edge 2019). Therefore, regulating these signaling pathways is likely to be an effective way to stimulate sensory progenitor cell proliferation and differentiation into new HCs. The Notch, Wnt, and Sonic hedgehog (SHH) signaling pathways play critical roles in controlling inner ear sensory cell proliferation and differentiation (Roccio and Edge 2019). The Notch signaling pathway induces supporting cell (SC) and HC proliferation and differentiation via lateral inhibition during the early development of the inner ear (Daudet and Zak 2020; Takebayashi et al. 2007), and the Wnt signaling pathway controls sensory progenitor cell polarity, proliferation, specification, and differentiation (van Amerongen and Nusse 2009). The SHH pathway plays an important role in inner ear development (Driver et al. 2008) and regulates cochlear HC differentiation (Benito-Gonzalez and Doetzlhofer 2014; Bok et al. 2013). The Notch and Wnt signaling pathways also crosstalk with each other in regulating and maintaining the balance between cell proliferation and differentiation in many tissues (Fre et al. 2009; Munnamalai and Fekete 2020). SHH and Notch function together to maintain various neuronal progenitor cell populations (Takanaga et al. 2009; Wu et al. 2012), and the interaction between Wnt and SHH signaling is important for the dorsal polarity of otocysts (Ohta and Schoenwolf 2018), and both pathways are required for the formation of the semicircular canals (Hwang et al. 2019). However, the crosstalk between the Notch, Wnt, and SHH signaling pathways in regulating the proliferation and regeneration of sensory progenitor cells in the mouse cochlea has not been reported in detail.

Therefore, we cultured neonatal mouse cochleae with DAPT, QS11, and/or SHH together with the cell proliferation marker EdU for 3 days. Also, we damaged the HCs with neomycin and then treated the cochleae with DAPT, QS11, and/or SHH for 7 days. Furthermore, using transgenic mice, we found that that Notch inhibition and Wnt activation could promote greater proliferation and regeneration of SCs and HCs compared to only inhibiting the Notch pathway. Moreover, simultaneously activating the Wnt and SHH signaling pathways could further collaborate with DAPT to promote the proliferation of sensory progenitor cells. Thus, our work presents novel information about the Notch, Wnt, and SHH signaling pathways and their crosstalk in regulating sensory cell proliferation and regeneration and suggests a new way to regenerate more mammalian HCs after they are damaged.

## Materials and methods

### Animals

Wild-type neonatal (postnatal day (P0–P4) C57BL/6j mice were from Fudan Medical School (Shanghai, China), and Atoh1-EGFP, Notch1-flox (exon1), and Catnb-flox (exon3) mice were purchased from the Jackson Laboratories. Sox2-Cre-ER mice were a gift from Dr. Konrad Hochedlinger of Harvard University. The care and use of animals were approved by the Institutional Animal Care and Use Committee of Fudan University in compliance with the NIH guidelines for the care and use of laboratory animals.

### Organotypic culture of wild-type mouse cochleae

The mice were euthanized by carbon dioxide asphyxiation and decapitated, and the heads were placed in 75% ethanol and quickly transferred to chilled Hanks’ balanced salt solution (HBSS). The temporal bones were dissected out, and the cochleae were isolated from the temporal bones using sterile procedures in ice-cold HBSS. The stria vascular and spiral ganglion were removed with fine forceps.

Explants of the organ of Corti were placed intact on polylysine-coated cover glasses (Sigma, St. Louis, MO, USA) and maintained in four-well culture dishes (Greiner Bio-One, Frickenhausen, Germany) in Dulbecco’s modified Eagle’s medium (DMEM) and F12 medium supplemented with N2 and B27 (Invitrogen/GIBCO/BRL, Carlsbad, CA, USA) and 50 IU/mL penicillin (Sigma, St. Louis, MO, USA). The tissues were incubated at 37 °C in a humidified atmosphere of 5% CO_2_.

### Treatment of cochlear cultures

Cultures were exposed to culture medium with 10% FBS for about 2 h and then given the following treatments: DAPT (γ-secretase inhibitor IX, *N*-[*N*-(3,5-difluorophenacetyl)-l-alanyl]-S-phenylglycinet-butylester, EMD, Gibbstown, NJ, USA); QS11 ((2S)-2-[2-(indan-5-yloxy)-9-(1,1′-biphenyl-4-yl) methyl]-9H–purin-6-ylamino)-3-phenyl-propan-1-ol, Tocris Biosciences, Ellisville, Missouri, USA), which modulates ARF-GTP levels and synergizes with the Wnt/β-catenin signaling pathway to upregulate β-catenin nuclear translocation; and recombinant SHH protein (R & D Systems, Minneapolis, MN, USA), which activates the SHH signaling pathway. All compounds were dissolved in sterile dimethylsulphoxide (DMSO, Sigma, St. Louis, MO, USA) to a certain concentration (DAPT 10 mM, QS11 10 mM, SHH 100 μg/ml) and stored in aliquots at –20 °C before dilution in culture medium to their final concentration immediately prior to use.

The cochlear explants were treated with 5 μM DAPT and 10 μM QS11 and/or 200 ng/ml SHH for 3 days. Control cultures were incubated in 0.1% DMSO or PBS. The cell proliferation marker EdU (5-ethynyl-2′-deoxyuridine, Invitrogen, Grand Island, NY, USA), which is efficiently incorporated into newly synthesized DNA, was added to the culture medium at a concentration of 10 μM for the entire culture period.

Another group of cochlear explants were treated with 1 mM neomycin (Sigma, St. Louis, MO, USA) for 16 h and then thoroughly rinsed in fresh medium. The cultures were then treated with 5 μM DAPT and 10 μM QS11 and/or 200 ng/ml SHH along with 10 μM EdU for another 7 days (Kersigo et al. 2021). Control cultures were cultured in 0.1% DMSO or PBS.

### Treatment of Notch1 knockout and β-catenin over-expression mouse cochleae

Sox2-CreER and Notch1-flox (exon1) mice were mated with Catnb-flox (exon3) mice to generate Sox2-CreER, Notch1-flox (exon1), and Catnb-flox (exon3) mice. Littermates without Notch1-flox (exon1) or without Catnb-flox (exon3) were used as controls. Tamoxifen (2 mg/25 g body weight, Sigma, St. Louis, MO, USA) was given to the female mice by intraperitoneal injection, and the mother transferred the tamoxifen to the pups via her milk to activate the Cre recombinase. EdU (50 mg/kg) was given to the pups by intraperitoneal injection twice a day for 7 days. The pups were sacrificed at 7 days, and their cochleae were dissected out for subsequent immunohistochemistry processing. The mice were genotyped after being sacrificed.

We also dissected the cochleae from P0 to P1 mice and cultured the cochleae. All of the pups were genotyped after being sacrificed. After 2 h of adhering, the cultured cochleae were exposed to 1.0 mM neomycin in the culture medium for 16 h. The cultures were washed thoroughly with fresh medium and then treated with 1 μM tamoxifen (Sigma, St. Louis, MO, USA) for 2 days and then incubated in culture medium containing 10 μM EdU for another 7 days.

### Immunohistochemistry

The cultured cochleae were harvested and fixed for 30 min at room temperature with 4% paraformaldehyde in 0.1 M phosphate buffer and then thoroughly rinsed with 0.01 M PBS. After being permeabilized in 0.5% Triton X-100 in PBS (PBST) for 30 min at room temperature, the proliferating cells were labeled with EdU (Click-iT EdU Alexa Fluor 488 Imaging Kit, Invitrogen, Carlsbad, CA, USA) for another 30 min according to the manufacturer’s protocol. The tissues were blocked with 10% horse serum in PBST for 30 min, and the HCs were labeled with the rabbit antibody against Myo7a (1:500 dilution; Proteus Biosciences, Ramona, CA, USA) and the goat polyclonal antibody against Sox2 (1:200 dilution; Santa Cruz Biotechnology, Santa Cruz, CA, USA) at 4 °C overnight.

After being washed with PBST to remove the unbound antibodies, the tissues were incubated with donkey antirabbit (1:500 dilution; Invitrogen, Grand Island, NY) and donkey antigoat (1:200 dilution; Invitrogen, Grand Island, NY) diluted in PBST for 1 h at room temperature to visualize Myo7a and Sox2, respectively. The tissues were stained with 4′, 6-diamidino-2-phenylindole dihydrochloride (DAPI) for 5 min at room temperature to visualize the cell nuclei.

To detect functional mechanoelectrical transduction channels, 5 μM FM1-43 (Thermo Fisher Scientific, Eugene, OR, USA) was incubated with the cultured organ of Corti for 30 s and then washed with PBS for three times before fixation.

### Image acquisition and quantification

The fluorescent antibody-labeled organ of Corti samples was visualized using a Nikon (Miyagi, Japan) Eclipse 80i microscope, and the high-magnification fluorescent images were obtained with a Leica TCS SP5 microscope (Wetzlar, Germany). Cells were counted manually with the ImageJ software (Wayne Rosband, NIH, USA). The whole-mount cochleae were split into the apical, middle, and basal turns for counting the EdU-labeled cells, Sox2-labeled cells, and Myo7a-labeled cells. All cells were counted per 100-μm length of the cochlea. The length of each region was measured by drawing a line between inner and outer HCs using the ImageJ software. At least five samples in each group from three independent experiments were collected for statistical analysis. The cell counts for the control and treated groups were compared using Student’s *t*-test.

### RNA extraction for RNA-Seq analysis

Ten cultured cochleae from independent culture groups were dissolved in RNALater, and RNA-Seq libraries were generated using the SMART-Seq® v4 Ultra® Low Input RNA Kit for Sequencing (Takara Bio USA, Mountain View, CA, USA). Quality control of the raw sequencing data was performed using FastQC (http://www.bioinformatics.babraham.ac.uk/). Adaptors were trimmed and low-quality reads were removed using Trimmomatic (Bolger et al. 2014). Alignment to the mouse reference genome (mm10) was done with Hisat2, and aligned reads were counted with HTseq (https://doi.org/10.1093/bioinformatics/btu638) according to the UCSC mm10 annotation. Differential expression analysis between conditions was performed using the DESeq2 R package (Love et al. 2014), and genes with an adjusted p-value < 0.05 and fold-change > 1.5 were considered to be differentially expressed. Gene ontology analysis of the significantly differentially expressed genes was done with DAVID, and pheatmap in the R package was used to generate heatmaps.

We also performed quantitative real-time PCR (qRT-PCR) to validate the differentially expressed genes. Three to five cultured cochleae from independent culture groups were pooled and dissolved in 500 μl TRIzol (Invitrogen), and the total RNA was extracted using the RNeasy Micro Kit (QIAGEN, Hilden, Germany) according to the manufacturer’s protocol. The mRNA was reverse-transcribed to synthesize cDNA using the GoScript™ Reverse Transcription System (Promega, Maddison, WI, USA). qRT-PCR was performed using the GoTaq® qPCR Master Mix (Promega, Maddison, WI, USA), 1 μl retrotranscribed cDNA, and gene-specific primer sets in a volume of 20 μl in a LightCycle 480 (Roche). GAPDH was used for calibration. Each qRT-PCR was performed in triplicate in a volume of 20 μL using a StepOne Plus Real-Time PCR System (Applied Biosystems, Foster City, CA) for 35 cycles. Gene expression levels in the samples of each group were calculated using the comparative CT method where ΔΔCT = ΔCT sample – ΔCT control and the fold change = 2^−ΔΔCT^.

### Statistics

All data were analyzed with the GraphPad Prism software using a two-tailed, unpaired Student’s *t*-test when comparing two groups or with a one-way ANOVA followed by a Dunnett’s multiple comparisons test when comparing more than two groups. All data are expressed as either a percentage or as the mean ± SEM. *p*-Values < 0.05 were considered statistically significant.

## Results

### Notch inhibition along with Wnt activation promotes SC proliferation and HC regeneration in the cochlea in vitro in an age-dependent manner

**Fig. 1:**
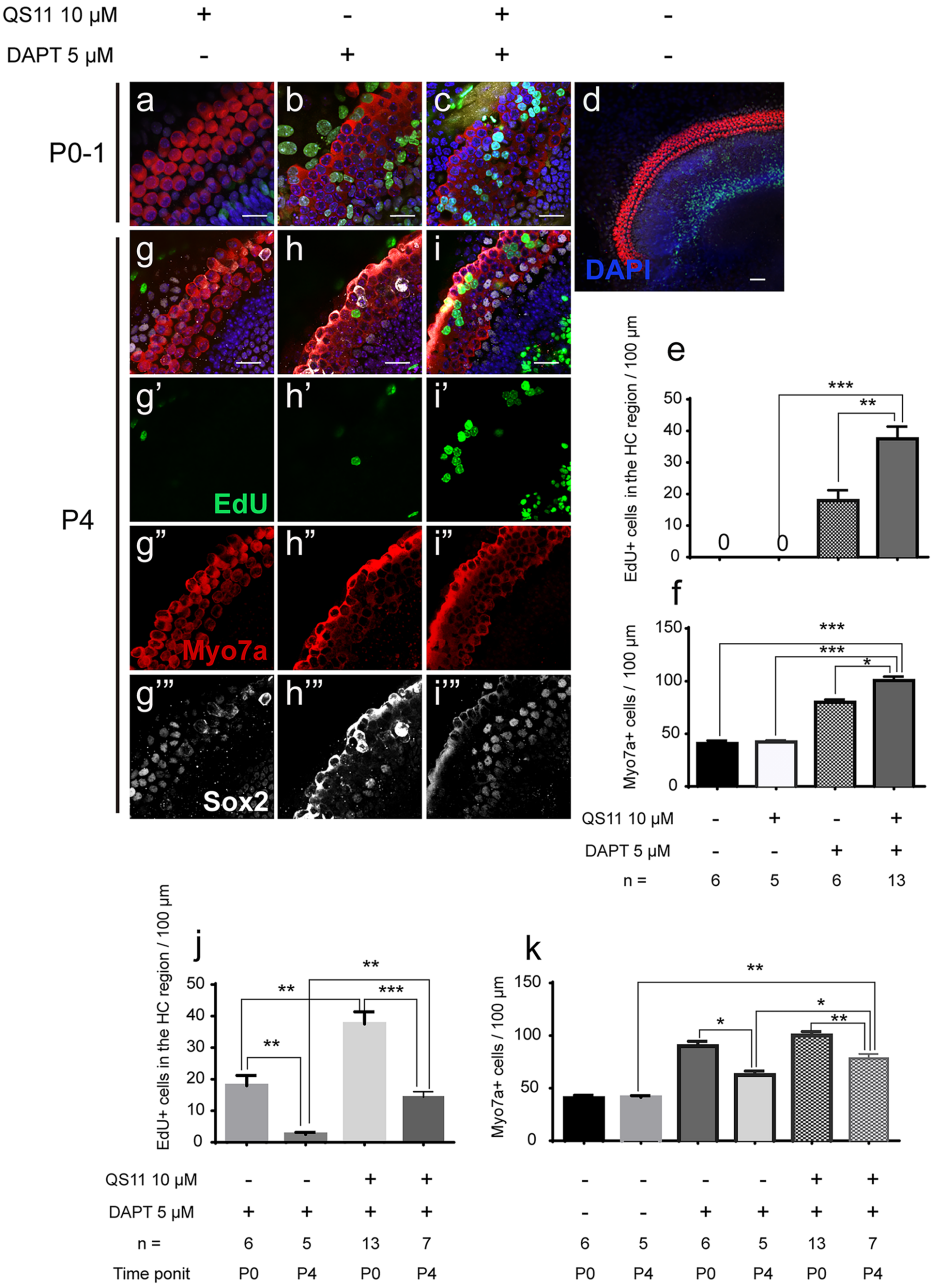
Co-treatment with DAPT and QS11 promotes SC proliferation and HC regeneration in the cochlea in vitro in an age-dependent manner. The P0–P1 cochleae were cultured with 10 μM QS11 **a** or 5 μM DAPT **b** or with both QS11 and DAPT **c** for 3 days. There were significantly greater numbers of EdU + /Sox2 + cells and EdU + /Myo7a + cells in the apex of the cochlea compared to the DAPT-only group and the QS11-only group. The EdU + cells in the DAPT + QS11 co-treated cochleae were seen throughout the sensory cell region in the apical turn. Scale bar = 20 μm. A control cochlea treated with vehicle is shown in **d** (Scale bar = 50 μm). The histograms show the differences in the numbers of EdU + cells **e** and Myo7a + cells **f** between these groups. The cells were counted per 100-μm length along the cochlea. The numbers of EdU + cells were counted between the outer HCs and the inner HCs. (*p < 0.05, **p < 0.01, ***p < 0.001). The P4 cochleae were cultured with 10 μM QS11 **g**–**g**’” or 5 μM DAPT **h**–**h**’” or with both QS11 and DAPT **i**–**i**’” for 3 days. The number of EdU + and Myo7a + cells was greater than in the DAPT-only and QS11-only groups, while the numbers of Myo7a + cells and EdU + cells in the sensory area were fewer compared to P0–P1 cochleae with the same treatment. Scale bar = 20 μm. The histograms show the differences in the numbers of Myo7a + cells **j** and EdU + cells **k** in the apical turn of the cochlea between the P0–P1 mice and the P4 mice for the different treatment groups. The cells were counted per 100-μm length along the cochlea. The numbers of EdU + cells were counted between the outer HCs and the inner HCs. (*p < 0.05, **p < 0.01, ***p < 0.001)

In the QS11-treated cochleae, there were no obvious EdU + /Sox2 + cells or EdU + /Myo7a + cells in the sensory epithelium, and the number of HCs did not change significantly (Fig. 1(a, d–f)). In the DAPT-only cochleae, numerous EdU + /Sox2 + cells and EdU + /Myo7a + cells were observed with a gradient from the apex to the base, with most cells seen in the apex, which was consistent with the literature (Matei et al. 2005). The number of HCs (HC_DAPT_ 80.09 ± 2.454/100 µm, N = 6) was significantly increased compared to the control group (HC_control_ 40.87 ± 2.692/100 µm, N = 6)(*p* < 0.05, DAPT vs control) (Fig. 1(b, d–f)). In the DAPT + QS11 co-treated cochleae, there were significantly more EdU + /Sox2 + cells and EdU + /Myo7a + cells (EdU cell_DAPT+QS11_ 37.51 ± 3.845/100 µm, N = 13) compared to the QS11-only group (EdU cell_QS11_ 0.0 ± 0.0, N = 5) (*p* < 0.001, DAPT + QS11 vs. QS11) and the DAPT-only group (EdU cell_DAPT_ 17.93 ± 3.252, N = 6) (*p* < 0.05, DAPT + QS11 vs. DAPT) (Fig. 1(c–f)). The EdU + cells of the co-treated group were spread throughout the HC region, and the number of HCs (HC_DAPT+QS11_ 100.6 ± 3.731/100 µm, N = 13) increased more than in the QS11-only (HC_QS11_ 42.24 ± 1.390/100 µm, N = 5) (*p* < 0.001, DAPT + QS11 vs. QS11) and the DAPT-only groups (*p* < 0.01, DAPT + QS11 vs. DAPT) (Fig. 1(a–c, e–f)).

We also used FM1-43 to label HCs with active mechanotransduction channels. FM1-43 fluorescence could be seen in the EdU + /Myo7a + cells in the cultured cochleae of the DAPT + QS11 group (Supplementary Fig. 1), suggesting that the mitotically regenerated HCs after DAPT and QS11 co-treatment had active mechanotransduction channels.

To further investigate whether Wnt upregulation and Notch inhibition could also induce cochlear SC proliferation and HC regeneration in older mice, we isolated P4 mouse cochleae and treated them with DAPT and/or QS11 and EdU. In the DAPT + QS11 group, there were also some EdU + /Sox2 + cells and EdU + /Myo7a + cells in the sensory cell region of the cochlea(EdU cell_DAPT+QS11_ 14.29 ± 1.769/100 µm, N = 7) (Fig. 1(i’–i’”)), and the number of EdU + cells was greater than in the DAPT-only group (EdU cell_DAPT_ 2.5 ± 0.646/100 µm, N = 5) (Fig. 1(h’–h’”, i’–i’”, j)) (*p* < 0.01, DAPT + QS11 vs. DAPT). The number of HCs also increased and was greater than the control and DAPT-only groups (HC_DAPT+QS11_ 78.25 ± 4.328/100 µm, N = 7; HC_control_ 41.33 ± 1.626/100 µm, N = 5; HC_DAPT_ 62.5 ± 3.797/100 µm, N = 5; *p* < 0.01, DAPT + QS11 vs. control; *p* < 0.05, DAPT + QS11 vs. DAPT) (Fig. 1(g’–g’”, h’–h’”, i’–i’”, k)). However, the number of EdU + cells and HCs in the P4 mice was lower than in the P0–P1 mice with the same treatment (for EdU + cells: p < 0.01, P4 vs. P0-P1 in DAPT-treated groups; p < 0.001, P4 vs. P0-P1 in DAPT + QS11-treated groups. For HCs: p < 0.05, P4 vs. P0-P1 in DAPT-treated group; p < 0.01, P4 vs. P0-P1 in DAPT + QS11-treated group) (Fig. 1(j, k)).

### Notch inhibition together with Wnt activation promotes SC proliferation and HC regeneration in damaged mouse cochleae in vitro

To investigate the effect of Wnt activation and Notch inhibition after HC ablation, we cultured P0–P1 mouse cochleae with neomycin to damage the sensory HCs. After HC ablation, the cochleae were treated with DAPT and/or QS11 and EdU for 7 days.

**Fig. 2:**
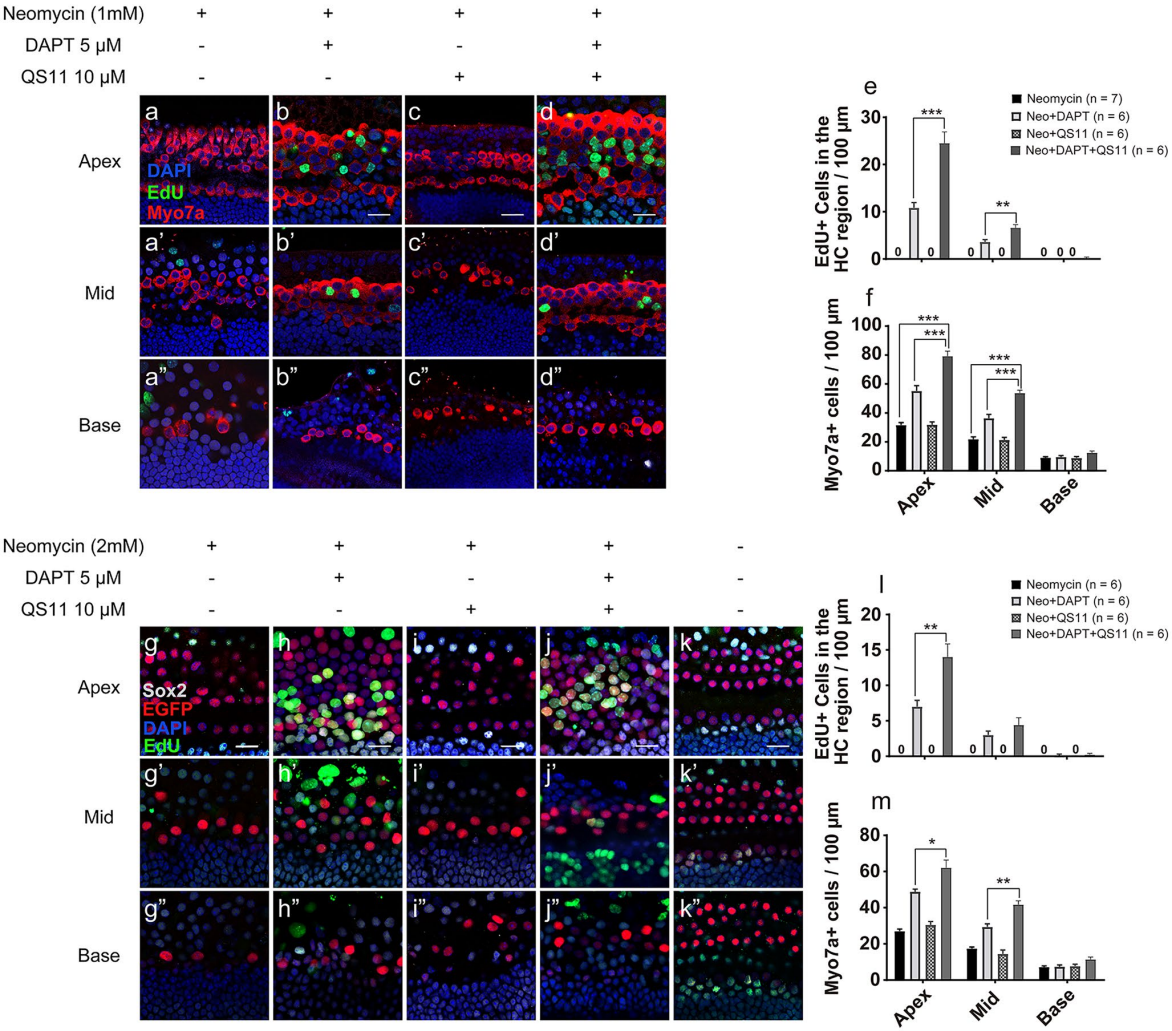
Co-treatment with DAPT and QS11 promotes SC proliferation and HC regeneration in neomycin-damaged cochleae in vitro. The P0–P1 cochleae were cultured with 1.0 mM neomycin for 16 h, then treated with either media only **a**–**a**”, 5 μM DAPT **b**–**b**”, 10 μM QS11 **c**–**c**”, or both **d**–**d**” for another 7 days. The number of Myo7a + cells was decreased in the apical, middle, and basal turns of the cochlea, especially in the basal turn and middle turns in the control and QS11-only groups. However, the numbers of EdU + /Sox2 + cells and EdU + /Myo7a + cells were increased in the DAPT-only and DAPT + QS11 groups, especially in the apical turn of the cochlea. In addition, there were many more EdU + cells in the sensory area of the cochlea than in the DAPT-only group, especially in the apical turn of the cochlea (scale bar = 20 μm). The histograms show the differences in the numbers of EdU + cells **e** and Myo7a + cells **f** in the different turns of the cochlea. The cells were counted per 100-µm length along the cochlea, which was measured between the outer HCs and the inner HCs (**p < 0.01, ***p < 0.001). The P0–P1 Atoh1-eGFP transgenic mouse cochleae were cultured with 2.0 mM neomycin for 16 h then treated with either media only **g**–**g**”, 5 μM DAPT **h**–**h**”, 10 μM QS11 **i**–**i**”, or both **j**–**j**” for another 7 days. Myo7a + cells were clearly lost in the basal turn and the middle turn of the cochlea in the control and QS11-only groups, and there were no EdU + /Sox2 + cells or EdU + /Myo7a + cells in the sensory domain in these two groups. However, there were some EdU + /Sox2 + cells and EdU + /Myo7a + cells in the sensory area of the cochleae in the DAPT-only and the combination groups, with more cells seen in the combination group compared to the DAPT-only group, especially in the apical turn of the cochlea. The P0–P1 Atoh1-eGFP mouse cochleae were cultured only with EdU, and there were no obvious EdU + /Sox2 + cells or EdU + /Myo7a + cells in the sensory area **k**. The histograms show the differences in the numbers of EdU + cells **l** and Myo7a + cells **m** in different turns of the cochlea. The cells were counted per 100-µm length along the cochlea, which was measured between the outer hair cells and the inner hair cells (*p < 0.05, **p < 0.01)

After being treated with 1 mM neomycin for 16 h, the DAPT-only group showed some EdU + /Sox2 + cells and a few EdU + /Sox2 + /Myo7a + triple-positive cells in the sensory epithelium region of the cochlea. The EdU + cells were mostly in the apex of the cochlea (EdU + cells_DAPT_ apex 10.8333 ± 1.108/100 µm, N = 6), there were a few EdU + cells in the middle turn of the cochlea, and there were no obvious EdU + cells in the base of the cochlea (EdU + cells_DAPT_ mid 3.600 ± 0.51/100 µm; base 0 ± 0.00/100 µm, N = 6). The number of HCs was increased (HC_DAPT_ apex 55.167 ± 3.646/100 µm; mid 36.333 ± 2.629/100 µm; base 9.500 ± 0.957/100 µm, N = 6. HC_Neo_ apex 31.600 ± 1.691/100 µm; mid 21.800 ± 1.655/100 µm; base 9.000 ± 0.707/100 µm, N = 7. DAPT vs neomycin, apex *p* < 0.001; mid *p* < 0.01; base *p* > 0.05), suggesting that DAPT had induced the regeneration of some new HCs (Fig. 2(a–a”, b–b”, e, f)).

In the DAPT + QS11 group, there were more EdU + /Sox2 + and EdU + /Sox2 + /Myo7a + triple-positive cells than in the DAPT-only group, and these EdU + cells were mostly found in the apical sensory epithelium region of the cochlea (EdU + cell_DAPT+QS11_: apex 24.500 ± 2.405/100 µm; mid 6.600 ± 0.678/100 µm; base 0.200 ± 0.200/100 µm, N = 6. DAPT + QS11 vs. DAPT, *p* < 0.01). There were also more newly regenerated HCs induced by DAPT + QS11 co-treatment compared to the DAPT-only group (HC_DAPT+QS11_: apex 79.167 ± 3.572/100 µm; mid 53.667 ± 2.044/100 µm; base 12.333 ± 1.333/100 µm, N = 6. DAPT + QS11 vs. DAPT, apex *p* < 0.001; mid *p* < 0.001; base *p* > 0.05) (Fig. 2(a–a”, b-b”, c–c”, d–d”, e, f)).

To investigate whether EdU + cells could be induced and whether new HCs could be regenerated in the sensory HC region after the HCs were even more heavily damaged, we treated the cultured cochleae with 2.0 mM neomycin for 24 h prior to treating the cochleae with 5 μM DAPT and/or 10 μM QS11 for 7 days. In the DAPT-only group and the DAPT + QS11 group, there were still some EdU + /Sox2 + cells and EdU + /Sox2 + /Myo7a + cells in the apical turn of the cochlea and a few cells in the middle turn (EdU + cell_DAPT+QS11_: apex 10.167 ± 1.276/100 µm; mid 2.000 ± 0.316/100 µm; base 0.000 ± 0.000/100 µm, N = 5. EdU + cell_DAPT_ apex 6.833 ± 1.057/100 µm; mid 1.600 ± 0.510/100 µm; base 0.000 ± 0.000/100 µm, N = 6.). Also, the number of EdU + /Sox2 + cells and EdU + /Sox2 + /Myo7a + cells in the DAPT + QS11 group was greater than in the DAPT-only group (*p* < 0.05, DAPT + QS11 vs. DAPT, EdU + cells in apex), and there were more newly regenerated HCs in the DAPT + QS11 group compared to the DAPT-only group (HC_DAPT+QS11_: apex 53.000 ± 3.066/100 µm; mid 31.333 ± 0.955/100 µm; base 10 ± 0.894/100 µm, N = 6. HC_DAPT_ apex 41.500 ± 1.057/100 µm; mid 24.5 ± 0.957/100 µm; base 7.167 ± 0.792/100 µm, N = 6. DAPT + QS11 vs. DAPT, *p* < 0.01, HCs in apex; *p* < 0.001, HCs in mid) (Supplement Fig. 2).

We also used Atoh1-eGFP mice to further confirm whether Notch inhibition and/or β-catenin upregulation could regenerate new HCs after the HCs were damaged. After exposure to 2.0 mM neomycin for 16 h, the cultured cochleae were treated with 5 μM DAPT and/or 10 μM QS11 for 7 days. In the DAPT-only group and the DAPT + QS11 group, we observed some EdU + /Sox2 + cells and EdU + /Sox2 + /Atoh1 − EGFP + triple-positive cells in the apical turn of the cochlea and a few in the middle turn (EdU + cell_DAPT+QS11_: apex 14.000 ± 1.844/100 µm; mid 4.400 ± 1.030/100 µm; base 0.200 ± 0.200/100 µm, N = 6. EdU + cell_DAPT_ apex 7.000 ± 0.894/100 µm; mid 3.000 ± 0.548/100 µm; base 0.143 ± 0.143/100 µm, N = 6) (Fig. 2(g–g”, h–h”, j–j”, k–k”, l)), and the number of HCs increased (HC_DAPT+QS11_: apex 62.000 ± 4.351/100 µm; mid 41.667 ± 2.124/100 µm; base 11.333 ± 1.358/100 µm, N = 6. HC_DAPT_ apex 48.333 ± 1.424/100 µm; mid 29.333 ± 1.726/100 µm; base 7.333 ± 0.989/100 µm, N = 6) (Fig. 2(g–g”, h–h”, j–j”, k–k”, m)). The number of EdU + cells and HCs induced by DAPT + QS11 was greater than that induced by DAPT-only treatment (DAPT + QS11 vs. DAPT, EdU + cells in the apex *p* < 0.01; HCs in the apex *p* < 0.05; HCs in the middle *p* < 0.01; HCs in the base *p* < 0.05) (Fig. 2(l–m)).

These results suggest that inhibiting Notch signaling induces regeneration of new HCs in the damaged cochlea and that simultaneous Notch inhibition and Wnt activation induces even more regenerated HCs in the damaged cochlea.

### Co-regulation of Notch inhibition and Wnt activation promotes SC proliferation and HC regeneration in transgenic mouse cochleae

**Fig. 3:**
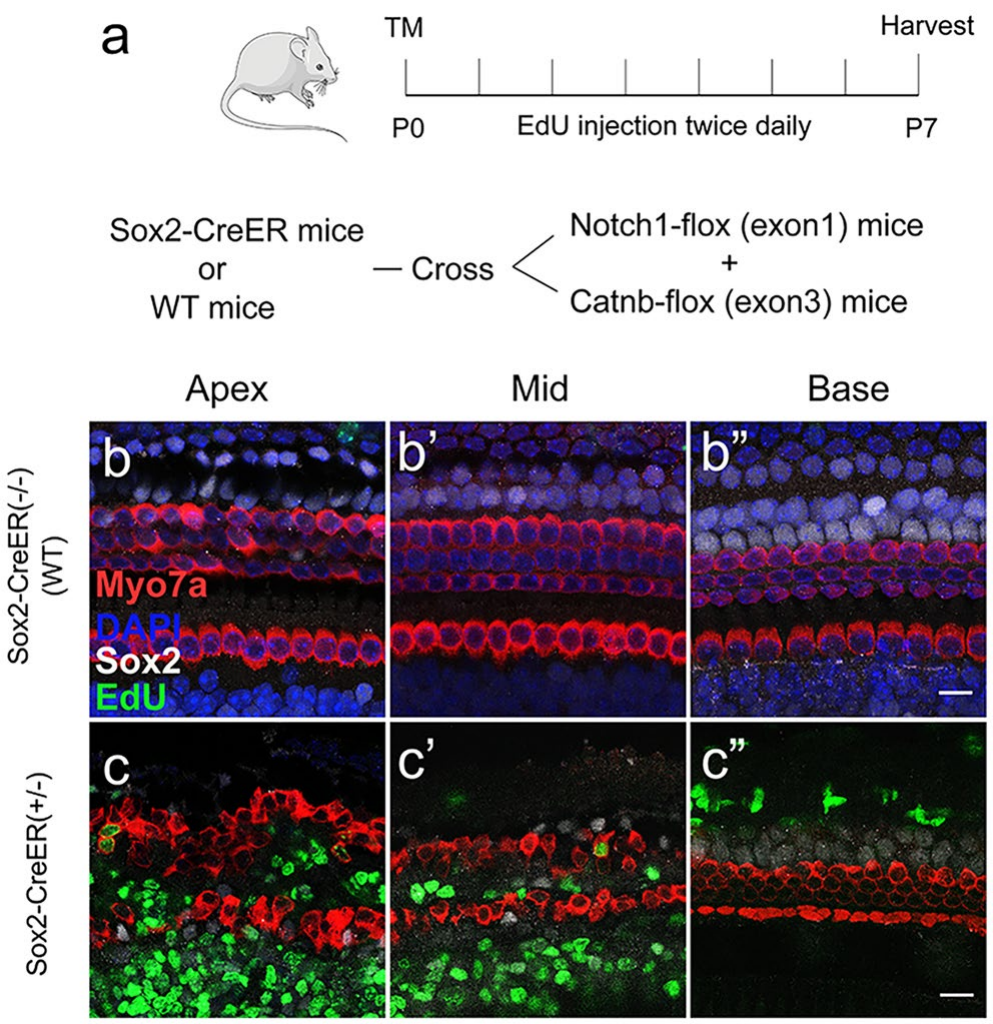
Transgenic mouse model for Notch1 inhibition and Wnt activation in SCs. The Sox2-CreER and Notch1-flox (exon1) mice were crossed with Catnb-flox (exon3) mice to generate pups. Tamoxifen (2 mg/25 g) was given to the female mice by intraperitoneal injection, and the mother transferred the tamoxifen to the pups via her milk to activate the Cre recombinase. EdU (50 mg/kg) was given to the pups by intraperitoneal injection twice a day for 7 days **a**. In the Notch1-flox (f/f), Catnb-flox (exon3) (f/ +), and Sox2-CreER (− / −) mouse cochleae, the number of Myo7a + cells was not changed and there were no obvious Sox2 + /EdU + or Myo7a + /EdU + cells in the sensory epithelium in the apical **b**, the middle **b**’, or the basal **b**” turns, while in the Notch1-flox (f/f), Catnb-flox (exon3) (f/ +), and Sox2-CreER (+ / −) mouse cochleae there were also numerous Sox2 + /EdU + cells, but few Sox2 + /EdU + cells, in the sensory epithelium. Most of the EdU + cells were in the apical turn and in the pillar cell region of the cochlea including the apical **c**, the middle **c**’, and the basal **c**” turns. Scale bar = 20 μm

We made the Sox2-CreER mice and Notch1-flox (exon1) mice and mated them with Catnb-flox (exon3) mice to generate Sox2-CreER, Notch1-flox (exon1), and Catnb-flox (exon3) mice in which Wnt signaling was activated and Notch signaling was inhibited specifically in Sox2 + SCs (Fig. 3(a)). In control mice lacking the Sox2-CreER line, the number of HCs was not significantly changed compared to wild-type mice and no Sox2 + /EdU + or Myo7a + /EdU + cells were observed in the sensory epithelium (Fig. 3(b–b”)). In the cochleae of the Notch1-flox (f/f), Catnb-flox (exon3) (f/ +), and Sox2-CreER (+ / −) mice, there were numerous Sox2 + /EdU + cells but only a few Myo7a + /EdU + cells in the sensory epithelium. Most of the EdU + cells were in the apical turn and in the pillar cell region of the cochlea. The number of HCs was increased, especially in the apical turn of the cochlea (Fig. 3(c–c”)). The vast majority of the increased HCs were also Sox2 + and EdU + , indicating that they were mitotically regenerated immature HCs.

### Co-regulation of Notch inhibition, Wnt activation, and SHH activation promotes the proliferation of cochlear sensory progenitor cells in vitro

**Fig. 4:**
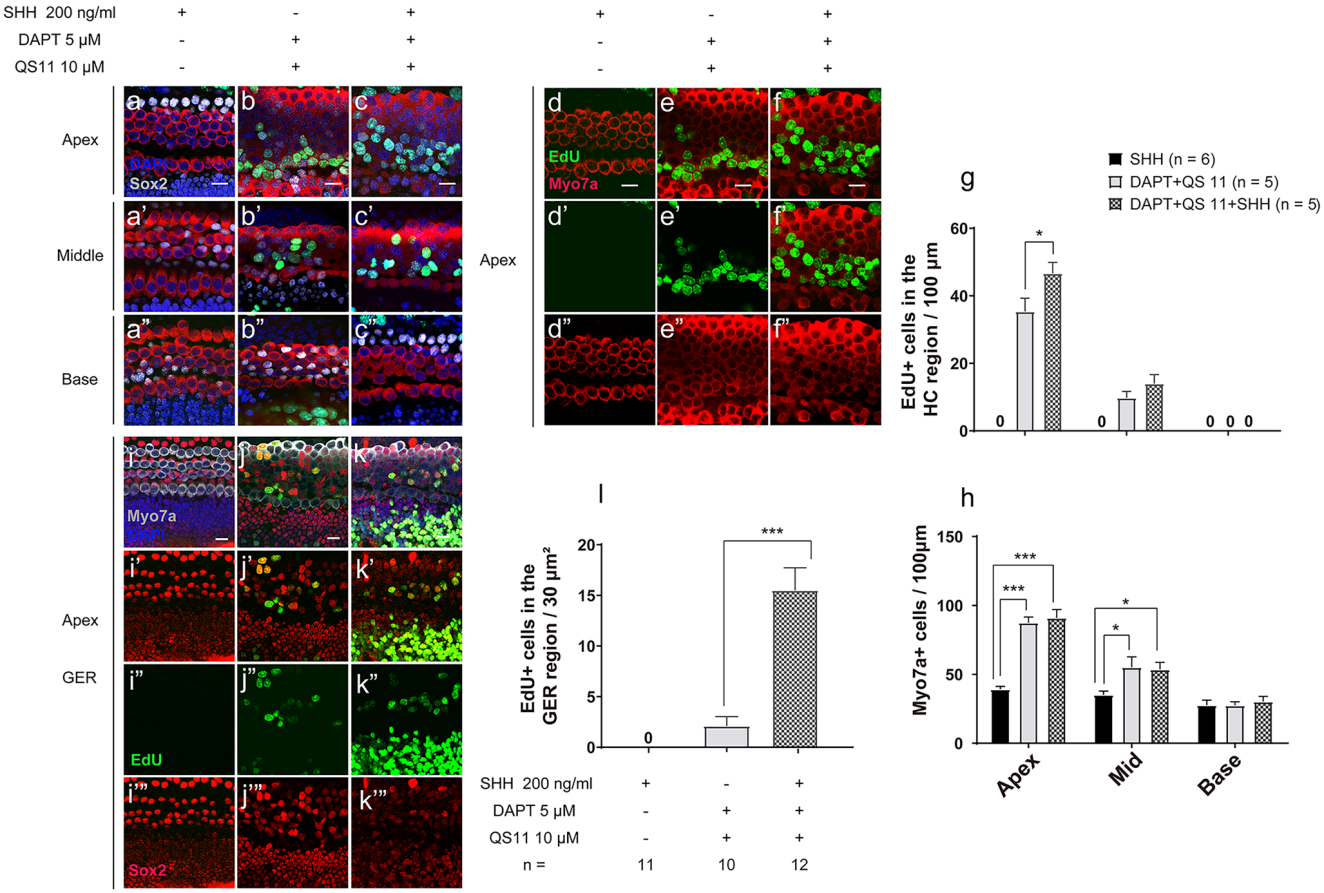
The role of SHH with DAPT and QS11 in the intact inner ear in vitro. Neonatal P0–P1 cochleae were treated with either 200 ng/ml SHH **a**–**a**”, **d**–**d**”, **i**–**i**’”, 5 μM DAPT, or 10 μM QS11 **b**–**b**”, **e**–**e**”, **j**–**j**’” or the combination of all three **c**–**c**”, **f**–**f**”, **k**–**k**’” for 3 days. There were EdU + /Sox2 + cells and EdU + /Myo7a + cells in the sensory area of the cochlea. The numbers of EdU + cells in the DAPT + QS11 + SHH group were much greater than those in the DAPT + QS11 group **a**–**a**”, **b**–**b**”, **c**–**c**”, especially in the apical turn of the cochlea **d**–**d**”, **e**–**e**”, **f**–**f**”. There were many more EdU + /Sox2 + cells in the GER of the cochlea in the DAPT + QS11 + SHH group compared to the other groups **i**–**i**’”, **j**–**j**’”, **k**–**k**’”. Scale bar = 20 μm. The histograms show the numbers of EdU + cells **g** and Myo7a + cells **h** in the sensory domain and EdU + /Sox2 + cells in the GER (l) in the different groups. The cells were counted per 100 μm length along the cochlea, which was measured between the outer hair cells and the inner hair cells (*p < 0.05, ***p < 0.001), or were counted per 30 μm^2^ area of the GER of the cochlea (*** *p* < 0.001)

P0–P1 mouse cochleae were treated with 5 μM DAPT, 10 μM QS11, and/or 200 ng/ml SHH for 3 days. In the SHH-only group, there were no obvious EdU + /Sox2 + cells or EdU + /Myo7a + cells in the sensory area of the cochlea (0.000 ± 0.000, N = 6), and the number of HCs showed no significant changes (apex 39.017 ± 2.249; mid 35.060 ± 2.735; base 27.500 ± 3.740, N = 6) (Fig. 4(a–a”, d–d”, g)). In the DAPT + QS11 + SHH group, there were many more EdU + /Sox2 + cells and EdU + /Myo7a + cells in the sensory area of the cochlea compared to the DAPT + QS11-treated group (EdU + cell_DAPT+QS11+SHH_: apex 46.667 ± 3.211/100 µm; mid 14.000 ± 2.646/100 µm; base 0.000 ± 0.000/100 µm, N = 5. EdU + cell_DAPT+QS11_: apex 35.333 ± 3.947/100 µm; mid 9.750 ± 1.931/100 µm; base 0.000 ± 0.000/100 µm, N = 5) (Fig. 4(b–b”, c–c”, e–e”, f–f”, h)), especially in the apical turn of the cochlea (DAPT + QS11 + SHH vs. DAPT + QS11, EdU + cells in apex p < 0.05) (Fig. 4(c–c”, f–f”, h)). The EdU + /Sox2 + cells in the DAPT + QS11 + SHH group appeared not only in the HC region, but also in the greater epithelial ridge (GER) of the cochlea, and there were more EdU + /Sox2 + cells in the GER compared to the DAPT + QS11-treated group (EdU + cell_DAPT+QS11+SHH_ 15.500 ± 2.221/100 µm, N = 12; EdU + cell_DAPT+QS11_ 2.100 ± 0.924/100 µm, N = 10; DAPT + QS11 + SHH vs. DAPT + QS11: *p* < 0.05) (Fig. 4(i–i’”, j–j’”, k–k’”, l)).

### Co-regulation of Notch inhibition, Wnt activation, and SHH activation also promotes sensory progenitor cell proliferation and HC regeneration in neomycin-damaged cochleae in vitro

**Fig. 5:**
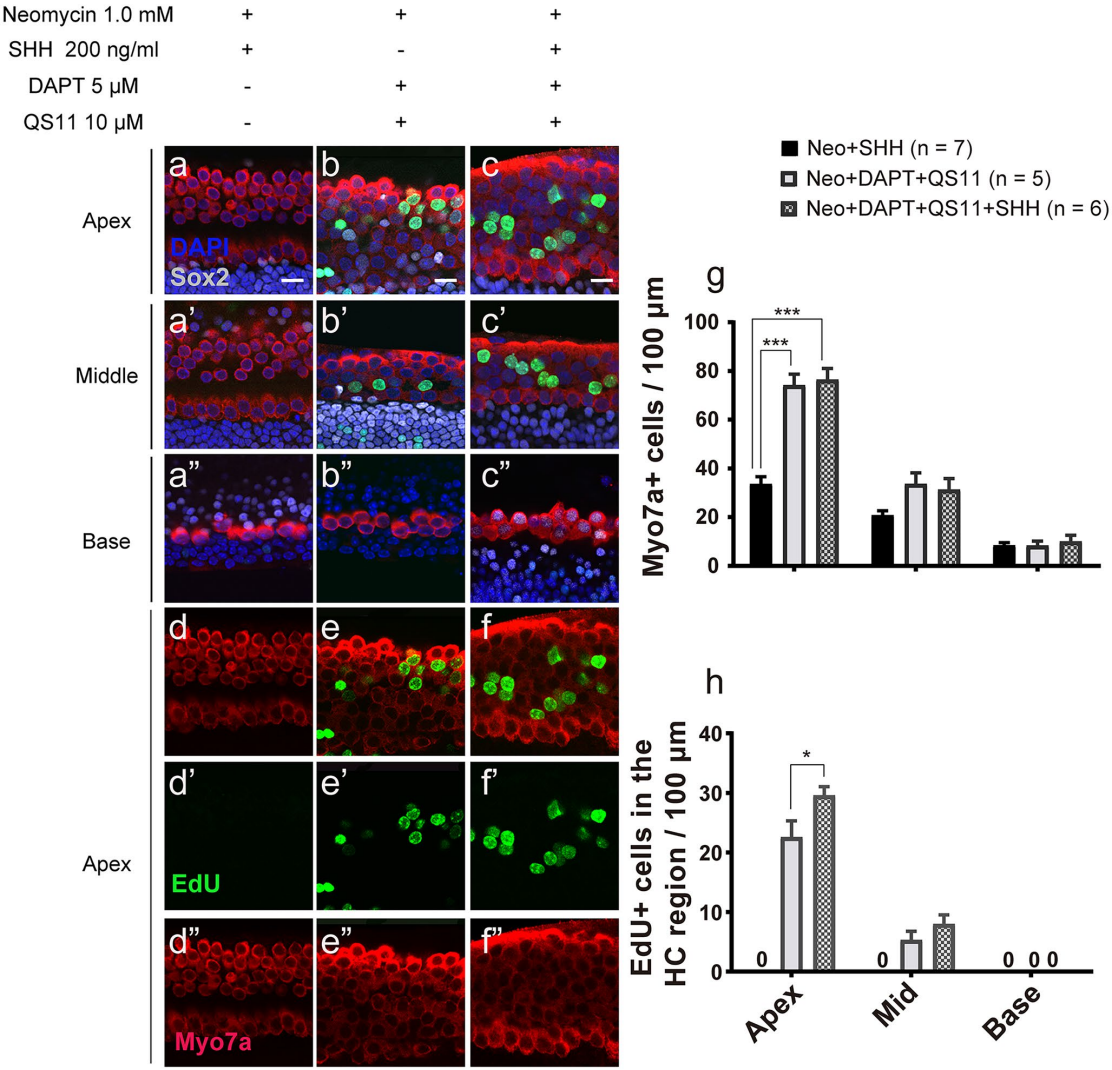
The role of SHH with DAPT and QS11 in the damaged inner ear in vitro. Neonatal P0–P1 cochleae were cultured with 1.0 mM neomycin for 16 h and then were treated with either 200 ng/ml SHH **a**–**a**”, **d**–**d**”, 5 μM DAPT, or 10 μM QS11 **b**–**b**”, **e**–**e**” or the combination of all three **c**–**c**”, **f**–**f**” for 7 days. There were some EdU + /Sox2 + cells and EdU + /Myo7a + cells in the sensory area of the cochlea. The numbers of EdU + cells in the DAPT + QS11 + SHH group were greater than those in the DAPT + QS11 group **a**–**a**”, **b**–**b**”, **c**–**c**”, especially in the apical turn of the cochlea **d**–**d**”, **e**–**e**”, **f**–**f**” (scale bar = 20 μm). The histograms show the numbers of Myo7a + cells **d** and EdU + cells **e** in these groups. The cells were counted per 100-μm length along the cochlea, which was measured between the outer hair cells and the inner hair cells (*p < 0.05, ***p < 0.001)

The P0–P1 mouse cochleae were treated with 1.0 mM neomycin for 16 h then given 5 μM DAPT, 10 μM QS11, and/or 200 ng/ml SHH for 7 days. In the DAPT + QS11-treated group and the DAPT + QS11 + SHH-treated group, there were some EdU + /Sox2 + cells and EdU + /Myo7a + cells in the sensory area of the cochlea (EdU + cell_DAPT+QS11+SHH_: apex 29.600 ± 1.435/100 µm; mid 8.000 ± 1.528/100 µm; base 0.000 ± 0.000/100 µm, N = 5. EdU + cell_DAPT+QS11_: apex 22.600 ± 2.713/100 µm; mid 5.333 ± 1.453/100 µm; base 0.000 ± 0.000/100 µm, N = 5), and the number of HCs increased, especially in the apical turn of the cochlea (HC_DAPT+QS11+SHH_: apex 76.500 ± 4.515/100 µm; mid 31.333 ± 4.470/100 µm; base 10.000 ± 2.517/100 µm, N = 6. HC_DAPT+QS11_: apex 74.167 ± 4.556/100 µm; mid 33.667 ± 4.440/100 µm; base 8.333 ± 1.764/100 µm, N = 5) (DAPT + QS11 + SHH vs. SHH, HCs in the apex *p* < 0.001) (Fig. 5(a–a”, b–b”, c–c”, d–d”, e–e”, f–f”, g)). The number of EdU + cells in the sensory area of the cochlea in the DAPT + QS11 + SHH-treated group was greater than that of the DAPT + QS11-treated group (DAPT + QS11 + SHH vs. DAPT + QS11, EdU + cells in the apex *p* < 0.05) (Fig. 5(h)).

### The mechanism by which DAPT + QS11 + SHH co-treatment induces SC and HC proliferation and regeneration in neomycin-damaged cochleae in vitro

**Fig. 6:**
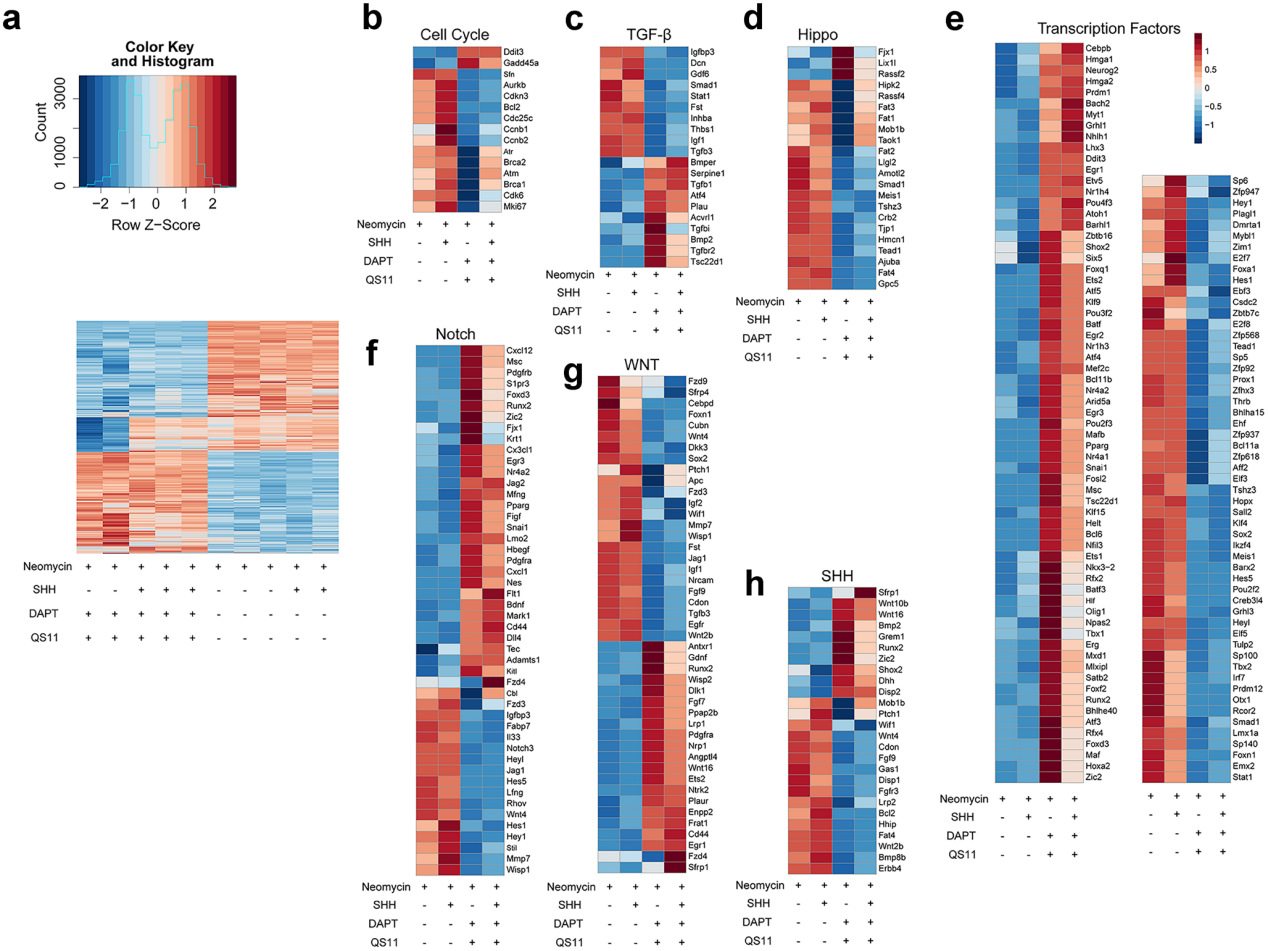
Gene expression and related mechanisms involved in SC proliferation and HC regeneration. DE genes in the neomycin control, Neo + SHH, Neo + DAPT + QS11, and Neo + DAPT + QS11 + SHH groups **a**. Red represents upregulated expression levels and blue represents downregulated expression levels. Each row represents one gene, and each column represents one experimental group. The heatmap shows the expression of DE genes involved in the cell cycle **b**, TGF-β **c**, Hippo **d**, transcription factor **e**, Notch **f**, Wnt **g**, and SHH **h** signaling pathways

To systematically investigate the molecular mechanism underlying the effect of DAPT + QS11 + SHH co-treatment, we performed RNA-Seq analyses to assess the genome-wide expression profiles in the cochleae from the neomycin-only control group, the Neo + SHH group, the Neo + DAPT + QS11 group, and the Neo + DAPT + QS11 + SHH group. A total of 3359 DE (differentially expressed) genes between each group were identified (Fig. 6a), and 429 DE genes between the Neo + DAPT + QS11 group and the Neo + DAPT + QS11 + SHH group were identified. Gene ontology analysis suggested the functional roles of these DE genes in sensory system development (Fig. 6b).

Among the DE genes between these groups, we found that the expression of many genes in the SHH, Hippo, Wnt, Notch, and TGF-β pathways as well as many transcription factors was not significantly different between the Neo + SHH group and the control group.

In the Neo + DAPT + QS11 group and Neo + DAPT + QS11 + SHH group compared to the control group, the upregulated genes included *Ddit3* and *Gadd45a* in the cell cycle pathway (Fig. 6b); *Bmper*, *Serpine1*, *Tgfb1*, *Bmp2*, and *Tgfbr2* in the TGF-β pathway (Fig. 6c); *Lix1l*, *Rassf2* in the Hippo pathway (Fig. 6d); *Pou4f3*, *Atoh1*, and *Pou3f2* in the transcription factor (TF) pathway (Fig. 6e); *Lrp1*, *Wnt16*, and *Cd44* in the Wnt pathway (Fig. 6g); and *Disp2* and *Bmp2* in the SHH pathway (Fig. 6h). The downregulated genes included *Notch3*, *Jag1*, *Hes5*, *Hes1*, *Heyl*, and *Hey1* in the Notch pathway (Fig. 6f); *Hipk2*, *Mob1b*, *Smad1*, and *Tead1* in the Hippo pathway (Fig. 6d); and *Ptch1* in the SHH pathway (Fig. 6h).

**Fig. 7:**
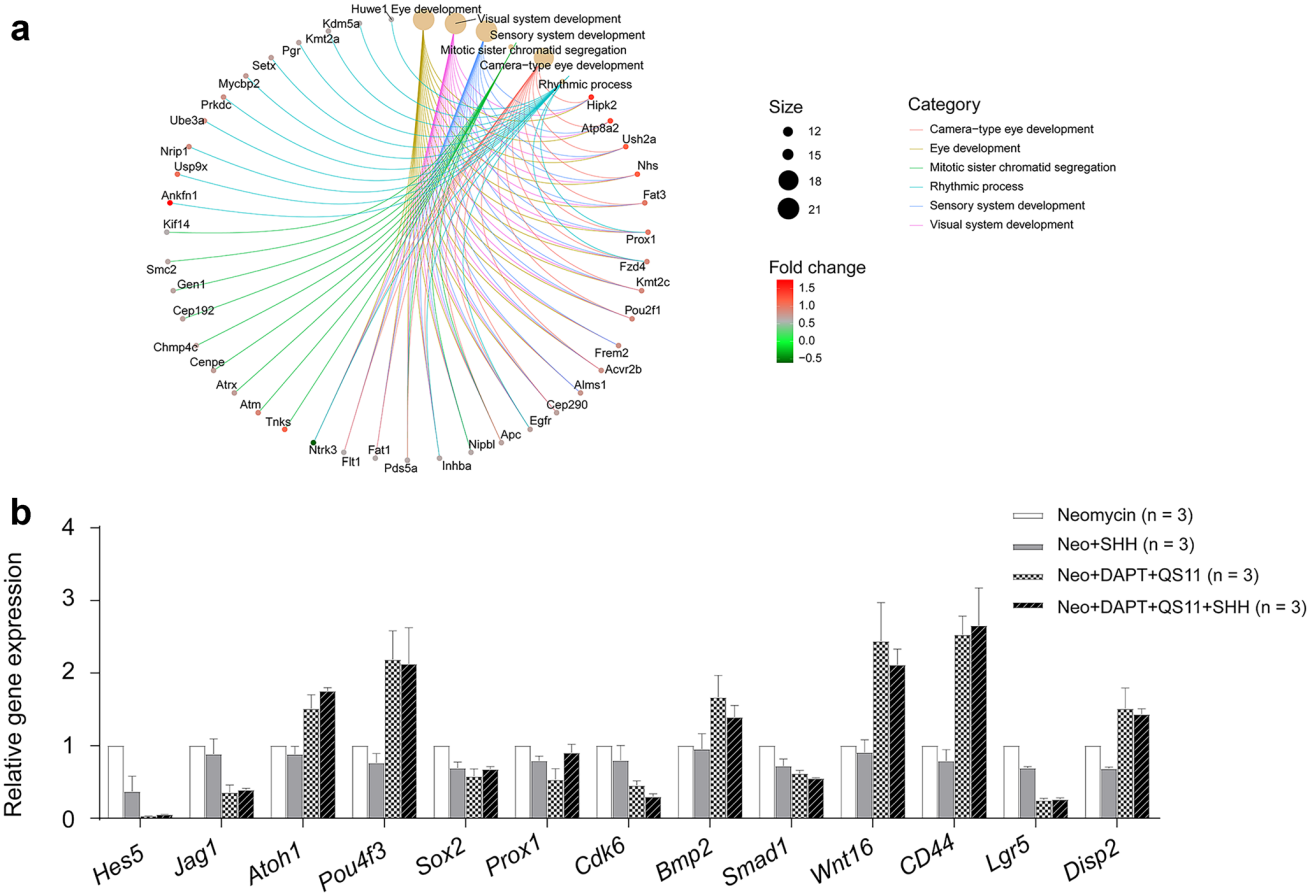
Differential expression analysis between groups. Gene ontology analysis was performed with the DE genes between the Neo + DAPT + QS11 and Neo + DAPT + QS11 + SHH groups **a**. The results of qRT-PCR of some of the DE genes showed differential expression in the Neomycin control, Neo + SHH, Neo + DAPT + QS11, and Neo + DAPT + QS11 + SHH groups, and these differences were consistent with the RNA-Seq results **b**

Comparing the Neo + DAPT + QS11 + SHH group and the Neo + DAPT + QS11 group, the upregulated genes included *Pou4f3* and *Atoh1* in the TF pathway (Fig. 6e); *Rassf4*, *Fat1*, *Fat3*, etc., in the Hippo pathway; *Cd44* and *Fzd4* in the Wnt pathway (Fig. 6g); and *Bmper*, *Serpine1*, and *Tgfb1* in the TGF-β pathway (Fig. 6c). The downregulated genes included *Hes1*, *Il33*, and *Fabp7* in the Notch pathway (Fig. 6f), *Wif1* in the SHH and Wnt pathways (Fig. 6g, h), and *Meis* and *Tshz3* in the Hippo pathway. These results indicated that treatment with SHH alone could not significantly upregulate the SHH pathway, while DAPT + QS11 co-treatment could. The DAPT + QS11 + SHH co-treatment induced even more SC proliferation, likely via the TF, Hippo, and Wnt signaling pathways in response to neomycin-induced damage in the cochlea (Fig. 7a).

The qRT-PCR also showed that the *Jag1*, *Hes1*, and *Hes5* genes were downregulated in the Neo + DAPT + QS11 and the Neo + DAPT + QS11 + SHH groups compared to the control group, while the *β-catenin*, *Wnt16*, *Cd44*, *Atoh1*, *Pou4f3*, *Bmp2*, and *Disp2* genes were upregulated (Fig. 7b). These results were consistent with the RNA-Seq results, which also indicated that Neo + DAPT + QS11 and Neo + DAPT + QS11 + SHH co-treatment could inhibit the Notch signaling pathway and activate the Wnt and SHH signaling pathways, while these pathways were unchanged in the SHH-only group.

## Discussion

Inner ear sensory HCs are required for hearing and balance functions. These highly differentiated mammalian sensory HCs are vulnerable to damage by ototoxic drugs, noise, genetic defects, etc., and they have a very limited ability to spontaneously regenerate new HCs after being damaged (Oesterle et al. 2008; Yamoah et al. 2020). In contrast, damaged HCs can be regenerated through the proliferation and differentiation of SCs in non-mammalian vertebrates (Hawkins et al. 2006). This ability is absent in mammals largely because the SCs cannot directly differentiate into HCs or undergo proliferation followed by differentiation into HCs. Therefore, controlling the genes and signaling pathways associated with HC regeneration in order to activate SC and HC proliferation and differentiation is the ideal way to induce HC regeneration.

### The orchestrated signaling pathways in cochlear development and HC regeneration

The Notch, Wnt, and SHH signaling pathways all participate in regulating the development of the inner ear. During inner ear development, the Notch signaling pathway regulates the directional differentiation of cells and the formation of chimeras (Daudet and Zak 2020; Yamamoto et al. 2006) and plays important roles in the process of HC regeneration. Inhibition of the Notch signaling pathway by γ-secretase inhibitors in newborn chickens and in neonatal and adult mice induces significant numbers of newly regenerated HCs that derive from sensory progenitor cells or SCs (Lin et al. 2011; Mizutari et al. 2013).

The Wnt signaling pathway also plays critical roles in inner ear development and is involved in otic placode directional differentiation, cochlear duct extension, sensory cell directional differentiation, and cell polarity regulation (Qian et al. 2007; van Amerongen and Nusse 2009). Activating the Wnt signaling pathway promotes the proliferation and colony formation of Lgr5 + sensory progenitor cells in the mouse cochlea (Chai et al. 2012).

SHH regulates the differentiation of HCs by influencing the cell cycle of the progenitor cells located in the cochlea (Bok et al. 2013), and it plays an important role in HC differentiation by regulating the expression of Atoh1 (Hu et al. 2010).

### Crosstalk between signaling pathways

The Notch and Wnt signaling pathways crosstalk with each other on all different levels in cells in a variety of tissues (Fre et al. 2009; Qian et al. 2015). β-catenin has a central position in the Wnt signaling pathway and is the most active component participating in the crosstalk with other pathways. Studies have shown that, in the inner ear, the Wnt signaling pathway can upregulate the expression of the *Jagged1*, *Notch1*, and *Hes1* genes, and these genes work through the classical Wnt/β-catenin signaling pathway to regulate the formation and size of the otic placode (Jayasena et al. 2008).

The Notch and SHH signaling pathways interact antagonistically to define the position and size of the prosensory domain in the developing cochlea (Driver et al. 2008), while the two pathways cooperate to maintain various neuronal stem and progenitor cell populations (Munnamalai and Fekete 2020; Wall et al. 2009). The balance between Wnt and SHH signaling activities is key in distinguishing between vestibular and auditory cell types (Hwang et al. 2019; Riccomagno et al. 2005).

The role of the crosstalk between all three of the Notch, Wnt, and SHH signaling pathways in sensory cell regeneration in the mammalian cochlea has not been reported before. In this study, we report the effect of the combination of Notch inhibition and Wnt activation and/or SHH signaling activation in sensory cell proliferation and regeneration in the damaged mouse cochlea. Notch pathway inhibition promotes progenitor cell differentiation into HCs in the inner ear (Jeon et al. 2011), while Wnt pathway activation promotes progenitor cell proliferation in the neonatal mouse cochlea (Chai et al. 2012). Our previous study found that inhibiting the Notch signaling pathway could induce SC proliferation and HC mitotic regeneration, while inhibiting the Wnt signaling pathway decreased SC proliferation and HC regeneration (Li et al. 2015). This means that the SC proliferation and HC regeneration induced by Notch inhibition are facilitated by activation of the Wnt signaling pathway.

In this study, we simultaneously inhibited the Notch signaling pathway and upregulated the Wnt signaling pathway in the mouse cochlea, and this induced much more SC proliferation compared to Notch inhibition alone, and the mitotically regenerated HCs with DAPT + QS11 co-treatment had active mechanotransduction channels. The same results were obtained in transgenic mice in which the Notch pathway was knocked out and the Wnt pathway was activated in Sox2 + SCs. With increased age, however, the proliferation and regeneration ability of the cochlear HCs and SCs induced by inhibition of Notch and activation of Wnt decreased, which may be due to the decreased expression of the Notch signaling pathway along with sensory HC differentiation (Lanford et al. 1999).

Notch signaling is upregulated after HC ablation in the mouse cochlea (Batts et al. 2009). Many previous studies have shown that Notch inhibition can generate new HCs by transdifferentiation of SCs in response to ototoxic damage (Lin et al. 2011). In our study, after HC ablation, simultaneous inhibition of Notch and activation of Wnt also promoted greater SC proliferation and HC regeneration compared to Notch inhibition alone. The experiments in the Atoh1-eGFP mouse cochlea further confirmed this result.

### The mechanism underlying the orchestrated signaling and gene expression

We performed RNA-Seq and analyzed the gene expression between the SHH-treated group, the DAPT + QS11-treated group, and the DAPT + QS11 + SHH-treated group in order to further explore the mechanism behind SC and HC proliferation and regeneration. When comparing the SHH-treated group with the control group, we found that gene expression between these two groups showed almost no differences. In contrast, 429 DE genes were identified when comparing the Neo + DAPT + QS11 group with the Neo + DAPT + QS11 + SHH group, including transcription factor genes, Hippo pathway genes, Wnt pathway genes, and TGF-β pathway genes. Thus, treatment with SHH could not significantly upregulate the SHH pathway, but co-treatment with DAPT + QS11 could upregulate the SHH pathway and induce SC proliferation and HC regeneration. Co-treatment with DAPT + QS11 + SHH induced even more SC proliferation via the transcription factor, Hippo, and Wnt signaling pathways.

These results suggest that the Wnt signaling pathway has synergistic effects with the Hippo signaling pathway in promoting SC proliferation and HC regeneration in the mouse cochlea, and simultaneous Notch inhibition and Wnt activation induced significant SC proliferation and led to the regeneration of numerous new HCs in the neonatal mouse cochlea. The new HCs were mostly regenerated from transdifferentiation of SCs, while some were mitotically regenerated from transdifferentiation of proliferating SCs and only a few were regenerated directly from mitotic division of surviving HCs. With the greater dose of neomycin and greater damage to the HCs of the cochlea, we observed more newly regenerated HCs by co-regulating the Notch and Wnt pathways compared to Notch inhibition alone. The sensory cells in the apical turn of the cochlea have a greater capacity for proliferation and mitotic generation compared to the middle and basal turns. Thus, the SC proliferation and HC regeneration were mostly seen in the apical turn of the mouse cochlea.

When co-regulating the Notch, Wnt, and SHH signaling pathways, the SHH pathway enhances the synergistic effects of the Notch and Wnt pathways to induce greater sensory progenitor cell proliferation not only in the HC area, but also in the GER of the cochlea. Some genes are involved in mediating the crosstalk between these signaling pathways (Chen et al. 2017), and the interaction of these signaling pathways promotes sensory progenitor cell proliferation and HC regeneration.

Some important genes involved in HC differentiation and regeneration, such as *p27*, *Hes1*, and *Hes5*, are expressed in the GER of the cochlea at different stages (Zheng et al. 2000; Zine et al. 2001). *Hes1* and *Hes5* are negative regulatory genes of HC differentiation and might participate in HC differentiation by antagonizing *Atoh1. Hes1* was mainly expressed in the GER and lesser epithelial ridge, while *Hes5* was mainly expressed in the lesser epithelial ridge and in only a narrow band of cells in the GER (Gemmell and Nelson 1992). *Hes5*-deficient mice have extra HCs, and the extra HCs also express *Atoh1* (Zine et al. 2001). In our study, co-regulating the Notch, Wnt, and SHH pathways downregulated *Hes1* and upregulated *Atoh1* more so than in the mice in which only Notch and Wnt pathways were co-regulated, and this might be the reason why there was greater sensory progenitor cell proliferation in the GER of the cochlea. The inner HCs might be derived from the cells on the farthest side of the GER (Gemmell and Nelson 1992). Taken together, our results suggest that co-regulating the Notch, Wnt, and SHH pathways is an effective way to induce more sensory progenitor cell proliferation that might lead to the regeneration of new inner HCs.

## Conclusions

In summary, we report that inhibition of the Notch signaling pathway and activation of the Wnt signaling pathway can induce SC proliferation and new HC regeneration after the HCs have been damaged and that this effect is further promoted by upregulation of the SHH signaling pathway. Thus, co-regulation of the Notch, Wnt, and SHH signaling pathways might provide a new and effective method for inducing sensory progenitor cell proliferation and HC regeneration after HC damage in the mammalian inner ear and for restoring hearing and balance functions.

## Supplementary Information

Below is the link to the electronic supplementary material.

Supplementary file1 (DOCX 383 KB)

## Data Availability

The datasets generated during or analyzed during the current study are available from the corresponding author on reasonable request.

## References

[CR1] Batts SA, Shoemaker CR, Raphael Y (2009). Notch signaling and Hes labeling in the normal and drug-damaged organ of Corti. Hear Res.

[CR2] Benito-Gonzalez A, Doetzlhofer A (2014). Hey1 and Hey2 control the spatial and temporal pattern of mammalian auditory hair cell differentiation downstream of Hedgehog signaling. The Journal of Neuroscience : the Official Journal of the Society for Neuroscience.

[CR3] Bok J, Zenczak C, Hwang CH, Wu DK (2013). Auditory ganglion source of Sonic hedgehog regulates timing of cell cycle exit and differentiation of mammalian cochlear hair cells. Proc Natl Acad Sci USA.

[CR4] Bolger AM, Lohse M, Usadel B (2014). Trimmomatic: a flexible trimmer for Illumina sequence data. Bioinformatics.

[CR5] Chai R, Kuo B, Wang T, Liaw EJ, Xia A, Jan TA, Liu Z, Taketo MM, Oghalai JS, Nusse R (2012). Wnt signaling induces proliferation of sensory precursors in the postnatal mouse cochlea. Proc Natl Acad Sci U S A.

[CR6] Chen Y, Lu X, Guo L, Ni W, Zhang Y, Zhao L, Wu L, Sun S, Zhang S, Tang M (2017). Hedgehog signaling promotes the proliferation and subsequent hair cell formation of progenitor cells in the neonatal mouse cochlea. Front Mol Neurosci.

[CR7] Daudet N, Zak M (2020). Notch signalling: the multitask manager of inner ear development and regeneration. Adv Exp Med Biol.

[CR8] Driver EC, Pryor SP, Hill P, Turner J, Ruther U, Biesecker LG, Griffith AJ, Kelley MW (2008). Hedgehog signaling regulates sensory cell formation and auditory function in mice and humans. The Journal of Neuroscience : the Official Journal of the Society for Neuroscience.

[CR9] Fre S, Pallavi SK, Huyghe M, Lae M, Janssen KP, Robine S, Artavanis-Tsakonas S, Louvard D (2009). Notch and Wnt signals cooperatively control cell proliferation and tumorigenesis in the intestine. Proc Natl Acad Sci USA.

[CR10] Gemmell RT, Nelson J (1992). Development of the vestibular and auditory system of the northern native cat, Dasyurus hallucatus. Anat Rec.

[CR11] Hawkins RD, Helms CA, Winston JB, Warchol ME, Lovett M (2006). Applying genomics to the avian inner ear: development of subtractive cDNA resources for exploring sensory function and hair cell regeneration. Genomics.

[CR12] Herranen A, Ikaheimo K, Lankinen T, Pakarinen E, Fritzsch B, Saarma M, Lindahl M, Pirvola U (2020). Deficiency of the ER-stress-regulator MANF triggers progressive outer hair cell death and hearing loss. Cell Death Dis.

[CR13] Hu X, Huang J, Feng L, Fukudome S, Hamajima Y, Lin J (2010). Sonic hedgehog (SHH) promotes the differentiation of mouse cochlear neural progenitors via the Math1-Brn3.1 signaling pathway in vitro. J Neurosci Res.

[CR14] Hwang CH, Keller J, Renner C, Ohta S, Wu DK (2019) Genetic interactions support an inhibitory relationship between bone morphogenetic protein 2 and netrin 1 during semicircular canal formation. Development *146* 10.1242/dev.174748 PMC6398446 30770380

[CR15] Jayasena CS, Ohyama T, Segil N, Groves AK (2008). Notch signaling augments the canonical Wnt pathway to specify the size of the otic placode. Development.

[CR16] Jeon SJ, Fujioka M, Kim SC, Edge AS (2011). Notch signaling alters sensory or neuronal cell fate specification of inner ear stem cells. The Journal of Neuroscience : the Official Journal of the Society for Neuroscience.

[CR17] Jung JY, Avenarius MR, Adamsky S, Alpert E, Feinstein E, Raphael Y (2013). siRNA targeting Hes5 augments hair cell regeneration in aminoglycoside-damaged mouse utricle. Mol Ther.

[CR18] Kersigo J, Gu L, Xu L, Pan N, Vijayakuma S, Jones T, Shibata SB, Fritzsch B, Hansen MR (2021). Effects of Neurod1 expression on mouse and human Schwannoma cells. Laryngoscope.

[CR19] Lanford PJ, Lan Y, Jiang R, Lindsell C, Weinmaster G, Gridley T, Kelley MW (1999). Notch signalling pathway mediates hair cell development in mammalian cochlea. Nat Genet.

[CR20] Li W, Wu J, Yang J, Sun S, Chai R, Chen ZY, Li H (2015). Notch inhibition induces mitotically generated hair cells in mammalian cochleae via activating the Wnt pathway. Proc Natl Acad Sci U S A.

[CR21] Lin V, Golub JS, Nguyen TB, Hume CR, Oesterle EC, Stone JS (2011). Inhibition of Notch activity promotes nonmitotic regeneration of hair cells in the adult mouse utricles. J Neurosci.

[CR22] Love MI, Huber W, Anders S. (2014) Moderated estimation of fold change and dispersion for RNA-seq data with DESeq2. Genome Biol 15(12):550. 10.1186/s13059-014-0550-8. PMID: 25516281 10.1186/s13059-014-0550-8 PMC4302049 25516281

[CR23] Matei V, Pauley S, Kaing S, Rowitch D, Beisel KW, Morris K, Feng F, Jones K, Lee J, Fritzsch B (2005). Smaller inner ear sensory epithelia in Neurog 1 null mice are related to earlier hair cell cycle exit. Developmental Dynamics : an Official Publication of the American Association of Anatomists.

[CR24] Mizutari K, Fujioka M, Hosoya M, Bramhall N, Okano HJ, Okano H, Edge AS (2013). Notch inhibition induces cochlear hair cell regeneration and recovery of hearing after acoustic trauma. Neuron.

[CR25] Munnamalai V, Fekete DM (2020). The acquisition of positional information across the radial axis of the cochlea. Developmental Dynamics : an Official Publication of the American Association of Anatomists.

[CR26] Nakano Y, Wiechert S, Fritzsch B, Banfi B (2020) Inhibition of a transcriptional repressor rescues hearing in a splicing factor-deficient mouse. Life Sci Alliance *3* 10.26508/lsa.202000841 PMC7652395 33087486

[CR27] Oesterle EC, Campbell S, Taylor RR, Forge A, Hume CR (2008). Sox2 and JAGGED1 expression in normal and drug-damaged adult mouse inner ear. J Assoc Res Otolaryngol.

[CR28] Ohta S, Schoenwolf GC (2018) Hearing crosstalk: the molecular conversation orchestrating inner ear dorsoventral patterning. Wiley Interdiscip Rev Dev Biol *7* 10.1002/wdev.302 PMC5746457 29024472

[CR29] Petrovic J, Galvez H, Neves J, Abello G, Giraldez F (2015). Differential regulation of Hes/Hey genes during inner ear development. Dev Neurobiol.

[CR30] Qian C, Liu F, Ye B, Zhang X, Liang Y, Yao J (2015). Notch4 promotes gastric cancer growth through activation of Wnt1/beta-catenin signaling. Mol Cell Biochem.

[CR31] Qian D, Jones C, Rzadzinska A, Mark S, Zhang X, Steel KP, Dai X, Chen P (2007). Wnt5a functions in planar cell polarity regulation in mice. Dev Biol.

[CR32] Riccomagno MM, Takada S, Epstein DJ (2005). Wnt-dependent regulation of inner ear morphogenesis is balanced by the opposing and supporting roles of Shh. Genes Dev.

[CR33] Roccio M, Edge ASB (2019) Inner ear organoids: new tools to understand neurosensory cell development, degeneration and regeneration. Development *146* 10.1242/dev.177188 PMC6765123 31477580

[CR34] Takanaga H, Tsuchida-Straeten N, Nishide K, Watanabe A, Aburatani H, Kondo T (2009). Gli2 is a novel regulator of sox2 expression in telencephalic neuroepithelial cells. Stem Cells.

[CR35] Takebayashi S, Yamamoto N, Yabe D, Fukuda H, Kojima K, Ito J, Honjo T (2007). Multiple roles of Notch signaling in cochlear development. Dev Biol.

[CR36] van Amerongen R, Nusse R (2009). Towards an integrated view of Wnt signaling in development. Development.

[CR37] Wall DS, Mears AJ, McNeill B, Mazerolle C, Thurig S, Wang Y, Kageyama R, Wallace VA (2009). Progenitor cell proliferation in the retina is dependent on Notch-independent Sonic hedgehog/Hes1 activity. J Cell Biol.

[CR38] Wu SM, Tan KS, Chen H, Beh TT, Yeo HC, Ng SK, Wei S, Lee DY, Choo AB, Chan KK (2012). Enhanced production of neuroprogenitors, dopaminergic neurons, and identification of target genes by overexpression of sonic hedgehog in human embryonic stem cells. Stem Cells Dev.

[CR39] Yamamoto N, Tanigaki K, Tsuji M, Yabe D, Ito J, Honjo T (2006). Inhibition of Notch/RBP-J signaling induces hair cell formation in neonate mouse cochleas. J Mol Med (berl).

[CR40] Yamoah EN, Li M, Shah A, Elliott KL, Cheah K, Xu PX, Phillips S, Young SM, Jr Eberl DF, Fritzsch B (2020) Using Sox2 to alleviate the hallmarks of age-related hearing loss. Ageing Res Rev *59* 101042 10.1016/j.arr.2020.101042 PMC7261488 32173536

[CR41] Zheng JL, Shou JY, Guillemot F, Kageyama R, Gao WQ (2000). Hes1 is a negative regulator of inner ear hair cell differentiation. Development.

[CR42] Zine A, Aubert A, Qiu J, Therianos S, Guillemot F, Kageyama R, de Ribaupierre F (2001). Hes1 and Hes5 activities are required for the normal development of the hair cells in the mammalian inner ear. The Journal of Neuroscience : the Official Journal of the Society for Neuroscience.

