# Supplementary material for: The crosstalk between the Notch, Wnt, and SHH signaling pathways in regulating the proliferation and regeneration of sensory progenitor cells in the mouse cochlea
Source: Cell Tissue Res. 2021 Jul 5;386(2):281–96. doi: 10.1007/s00441-021-03493-w (PMC8557196; doi:10.1007/s00441-021-03493-w)
Supplement: Supplementary file 1 — Supplementary file1 (DOCX 383 KB) [file 441_2021_3493_MOESM1_ESM.docx]

**Supplementary Figure:**


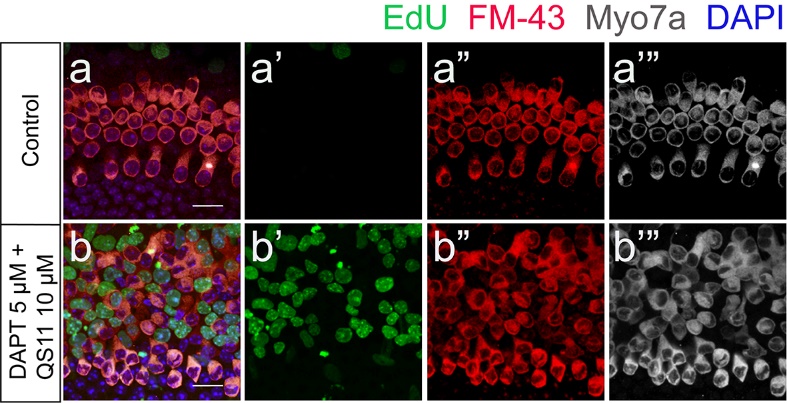


**Supplementary Figure 1**: The control group P0–P1 cochleae were cultured only with EdU (l–l3) or with both 5 μM DAPT and 10 μM QS11 (m–m3) for 3 days. There were no obvious EdU+/Myo7a+ cells in the sensory area, while all the Myo7a+ cells were colocalized with FM-43, which indicated the mechanotransduction channels in the HCs (Scale bar = 20 μm).


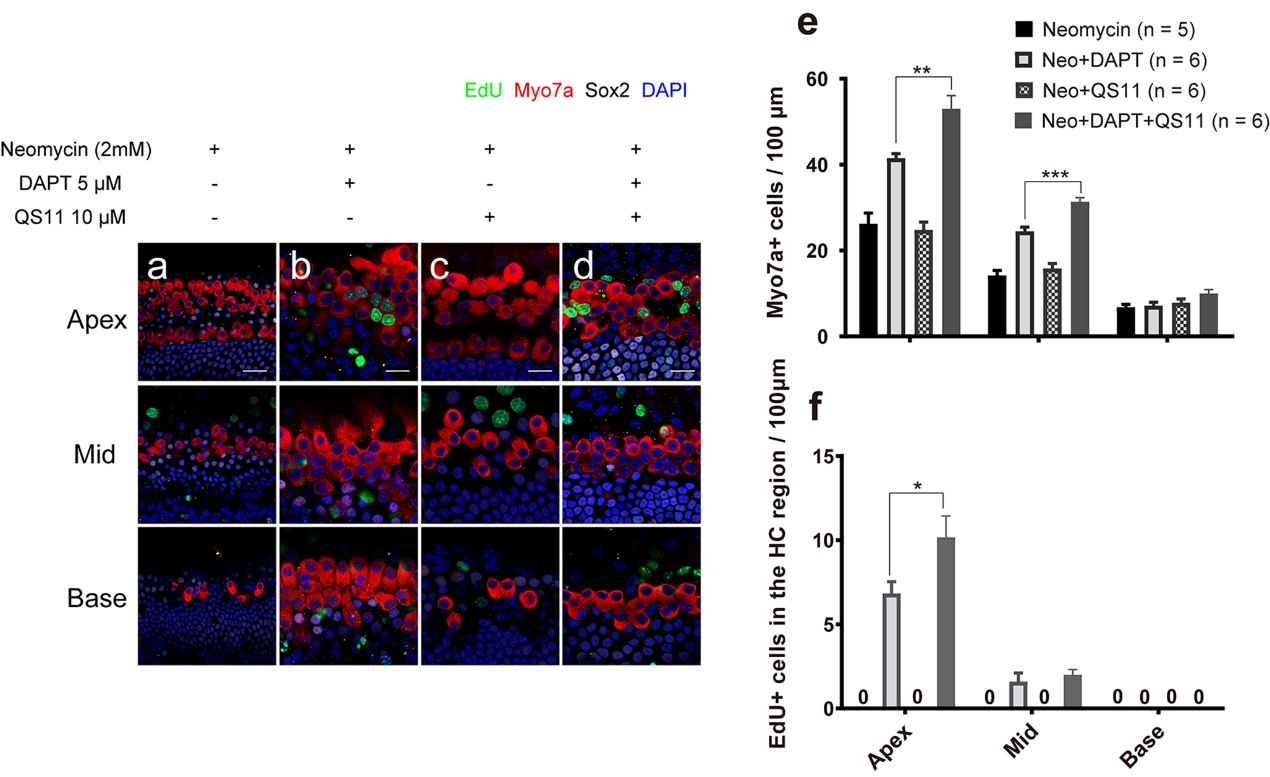


**Supplementary Figure 2: Lower regeneration capacity of progenitors by Notch inhibition and Wnt activation in cochleae treated with high concentrations of neomycin.**

The neonatal P0–P1 mouse cochleae were treated with 2.0 mM neomycin for 24 hours and then treated with media alone (a), 5 μM DAPT (b), 10 μM QS11 (c), or both (d) for another 7 days. The number of HCs was decreased in the apical, middle, and basal turns of the cochlea, especially in the basal turn and the middle turn in the neomycin-only and Neo+QS11 groups. There were still some EdU+/Sox2+ cells and EdU+/Myo7a+ cells in the sensory area of the cochlea, especially in the apical turn of the cochlea in the other two groups. Furthermore, there were many more EdU+ cells in the sensory area of the DAPT+QS11 cochlea compared to the DAPT-only group, especially in the apical turn of the cochlea.

The histograms show the differences in the numbers of EdU+ cells (e) and Myo7a+ cells (f) in different turns of cochlea between the groups. The cells were counted per 100 μm length along the cochlea, which was measured between the outer hair cells and the inner hair cells. (**p* < 0.05, ***p* < 0.01, ****p* < 0.001).
